# Peanut butter confirmed as the source in a case of infant botulism, United Kingdom, 2024

**DOI:** 10.2807/1560-7917.ES.2025.30.30.2500512

**Published:** 2025-07-31

**Authors:** Rosie J Crane, Corinne FL Amar, Humphrey Omoruyi, Burhan Ahmed, Jonathan Finch, Rachael Kendrick, Shamez Ladhani, Anais Painset, Dunstan Rajendram, Nika Raphaely, Buvana Dwarakanathan, Suba Guruprasad, Hani Zuhur-Adi, Vanessa K Wong, Gauri Godbole

**Affiliations:** 1 St George's University Hospitals NHS Foundation Trust, London, United Kingdom; 2 United Kingdom Health Security Agency (UKHSA), London, United Kingdom; 3 United Kingdom Health Security Agency (UKHSA), Kent Surrey Sussex, United Kingdom; 4 Royal Surrey County Hospital NHS Foundation Trust, United Kingdom

**Keywords:** Infant botulism, peanut, Clostridium botulinum, allergy

## Abstract

A 6-month-old infant was hospitalised with suspected infant botulism after being given peanut butter to reduce their risk of developing peanut allergy. *Clostridium botulinum* type A was detected in their faeces and the peanut butter by PCR and culture. Whole genome sequencing confirmed identical strains, identifying the peanut butter as the source of infection. The infant was treated with human-derived antitoxin Botulism Immune Globulin Intravenous (Human) (BIG-IV) and recovered well. This case highlights potential botulism risk with early peanut introduction.

We present a confirmed case of infant botulism linked to peanut butter that occurred in the United Kingdom in May 2024. The infant was fed peanut butter from 6 months of age following national clinical guidance, first issued in 2018, that aims to reduce risk of development of peanut allergy. Nuts may become contaminated with *Clostridium botulinum* spores at agricultural source or during processing. Here, we describe the case and discuss its implications for public health.

## Case detection and description

A previously healthy 6-month-old infant presented with a 1-day history of progressive lethargy, poor feeding, hoarse cry and noisy breathing, following 2 weeks of constipation. The infant had recently started eating solids, having previously been exclusively breastfed from birth. They had exposure to dust and soil playing in the garden. Blood and urine cultures, chest X-ray and blood inflammatory markers were unremarkable. Adenovirus and rhinovirus were detected in a nose/throat swab.

The infant was treated initially with ceftriaxone for suspected sepsis. By day 5 of illness, their lethargy had not improved. They developed head lag, poor swallowing, stridor, hyporeflexia and bilateral ptosis. A computed tomography (CT) scan of the brain was normal. Cerebrospinal fluid (CSF) had normal cytology and was culture-negative. Magnetic resonance imaging (MRI) of the brain was normal, and electromyography revealed reduced motor unit recruitment and decreased compound muscle action potentials, suggesting a presynaptic disorder. Botulism was considered likely and human-derived *C. botulinum* antitoxin (BIG-IV) was ordered on day 5 following discussion with the Infant Botulism Treatment and Prevention Programme in the United States [1]. The infant required intubation and ventilation on day 6 due to worsening hypoventilation resulting in respiratory failure.

On day 7, faeces of the infant were referred to the *C. botulinum* reference laboratory at the United Kingdom Health Security Agency (UKHSA). *Clostridium botulinum* type A was detected by PCR the next day and subsequently on culture, confirming the clinical diagnosis of infant botulism.

BIG-IV was received and administered on day 10. The infant was extubated on day 20 but required non-invasive respiratory support until day 30. Intensive physiotherapy aided recovery, and the infant was discharged on day 44 with residual head lag and truncal instability. By day 65, muscular tone and power had normalised, but constipation persisted. By day 110, the infant had developed severe constipation, treatment of which was challenging due to their aversion to oral medications. Constipation had greatly improved by day 140 and resolved by day 234.

## Source investigation

A review of the infant’s food history revealed that they had been fed incremental doses of a commercially prepared peanut butter, starting 10 days before onset of symptoms. The used container was referred for analysis to the reference laboratory, and *C. botulinum* type A was detected by PCR and isolated through culture. Whole genome sequencing single nucleotide polymorphism analysis (data not shown) confirmed that the isolates detected in the infant and the peanut butter were identical, which is consistent with the peanut butter being the source of infection. The product was made of roasted peanuts without addition of honey. Other consumed foods, including cooked and raw vegetables, washed fruit, yoghurt, cream cheese and cooked eggs, were either not suspected as being at-risk foods or not available and were not tested.

## Discussion

Infant botulism is a rare disease in the UK with only 23 confirmed cases since it was first recognised in the US in 1977 [2]. It primarily affects infants under 12 months of age and results from ingestion or inhalation of *C. botulinum* spores which then germinate in the immature gut, producing botulism neurotoxin (BoNT) in vivo. Ingestion of as few as 10 spores has led to disease in a mouse model [3]. More recently, intestinal botulism in an adult followed ingestion of a food source with just 14 spores per kilogram [4]. Spore count was not performed on the peanut butter in this case.

The BoNT blocks neurotransmitter release at neuromuscular junctions, leading to constipation and ‘floppy baby syndrome’ characterised by progressive symmetrical weakness, poor feeding, secretion pooling, ptosis, hyporeflexia and respiratory failure [5]. Early signs of botulism resemble common paediatric illnesses, with botulism often only suspected once symptoms have progressed. Broad-spectrum antibiotics are sometimes given on initial presentation when sepsis or specific infections are suspected. Bacterial infection should be ruled out early to facilitate early stopping of antibiotics, as the latter can induce rapid worsening of botulism symptoms due to BoNT release following *C. botulinum* cell lysis.

In the UK, infant botulism is confirmed by the detection of *C. botulinum* through culture and molecular testing of faeces or rectal washout. However, awareness of rectal washout as a sampling option is low in the UK, leading to delays as constipation often means faeces are not immediately available. Treatment involves the human-derived antitoxin BIG-IV, currently only available from the Infant Botulism Treatment and Prevention Programme in the US [6]. Most cases require intensive supportive care including respiratory support and prolonged hospitalisation.

Botulinum spores are ubiquitous, and the source of infant botulism is rarely identified. In the UK, cases have been microbiologically linked to honey, formula milk and terrapin pets [7]. Dust and soil are known to contain *C. botulinum* spores but are not usually sampled in individual clinical cases due to large coverage and low yield. Other risk factors in UK cases have included weaning, long distance travel, exposure to dust, and visits to petting zoos (data not shown).

This case of infant botulism was microbiologically and epidemiologically linked to the consumption of peanut butter. A similar case of intestinal botulism in an immunosuppressed adult following peanut butter ingestion was previously described in Canada where a subsequent regional survey found *C. botulinum* spores in 3% of peanut butter samples [4,8]. Raw peanuts harbour environmental spores which are heat resistant and can contaminate the peanut butter during food processing.

We did not test the peanut butter for BoNT. However, we would not expect it to have been present as the slow progression of symptoms was clinically consistent with sporulation and toxin production in the infant’s intestinal tract rather than preformed toxin causing food- botulism. There were no other reported cases of food botulism notified in England at the time.

High-quality international randomised trials have shown that introduction of peanuts into the diets of at-risk infants can help prevent peanut allergy later in life [9,10]. In 2018, the UK Scientific Advisory Committee on Nutrition and the Committee on Toxicity of Chemicals in Food, Consumer Products and the Environment recommended including peanuts and other allergenic foods in infants’ diets without delay once complementary foods have been introduced at around 6 months of age [11]. The British Society of Allergy and Clinical Immunology further advises, for babies at higher risk of allergy (defined as babies with severe eczema or pre-existing non-peanut food allergy), introduction of peanuts alongside complementary foods from 4 months of age onwards if the infant is deemed developmentally ready for solid foods [12]. This is in line with recommendations from other international societies [8]. In our reported case, the infant’s father had a severe peanut allergy, and the parents chose to introduce peanut butter from 6 months of age.

Peanut and other food allergies can be severe and life-threatening. Around 2% of children in the UK are affected by nut allergy [13]. In contrast, infant botulism is very rare. However, as more caregivers follow guidance to introduce nuts and seeds early in infants’ diets, more babies are likely to be exposed to spore-bearing foods, potentially posing an unrecognised botulism risk. 

## Conclusion

This case of infant botulism from peanut butter underscores the need for awareness of potential food-borne sources of *Clostridium botulinum* spores, particularly as more infants are fed allergenic foods early to reduce the risk of severe allergies later in life. Further awareness and safety considerations may be needed to balance allergy prevention with food-borne infection risks in infants.

## Data Availability

Depositing of the sequences generated in this investigation is pending. An addendum will be published here once they are available. Sequences generated in connection with this work are accessible at the National Center for Biotechnology Information (BioProject no. PRJNA1307529) under accession numbers 01579364 - SRR35023806, 01579365 - SRR35023805 and 01579366 - SRR35023804.^†^

## ^†^Addendum

The GenBank accession numbers of the genomic sequences obtained in the study were added to the Data availability statement on 21 August 2025.
