# Addendum for Euro Surveill. 2025;30(30)

**DOI:** 10.2807/1560-7917.ES.2025.30.33.250821a

**Published:** 2025-08-21

**Authors:** Eurosurveillance editorial team

**Affiliations:** 1 European Centre for Disease Prevention and Control (ECDC), Stockholm, Sweden

In the article ‘Peanut butter confirmed as the source in a case of infant botulism, United Kingdom, 2024’ by Crane et al. published in *Eurosurveillance* on 31 July 2025, the GenBank accession numbers of the genomic sequences obtained in the study were added to the Data availability statement on 21 August 2025.

